# Whole-exome sequencing in obsessive-compulsive disorder identifies rare mutations in immunological and neurodevelopmental pathways

**DOI:** 10.1038/tp.2016.30

**Published:** 2016-03-29

**Authors:** C Cappi, H Brentani, L Lima, S J Sanders, G Zai, B J Diniz, V N S Reis, A G Hounie, M Conceição do Rosário, D Mariani, G L Requena, R Puga, F L Souza-Duran, R G Shavitt, D L Pauls, E C Miguel, T V Fernandez

**Affiliations:** 1 Department of Psychiatry, University of São Paulo School of Medicine, Ovideo Pires de Campos, São Paulo, Brazil; 2 Department of Psychiatry, University of California, San Francisco, San Francisco, CA, USA; 3 Neurogenetics Section, Centre for Addiction and Mental Health, Institute of Medical Science and Department of Psychiatry, University of Toronto, Toronto, ON, Canada; 4 Federal University of São Paulo-UPIA-UNIFESP, São Paulo, Brazil; 5 Department of Psychiatry, Harvard Medical School, Psychiatric and Neurodevelopmental Genetics Unit, Center for Human Genetics Research, Massachusetts General Hospital, Boston, MA, USA; 6 Child Study Center and Department of Psychiatry, Yale University School of Medicine, New Haven, CT, USA

## Abstract

Studies of rare genetic variation have identified molecular pathways conferring risk for developmental neuropsychiatric disorders. To date, no published whole-exome sequencing studies have been reported in obsessive-compulsive disorder (OCD). We sequenced all the genome coding regions in 20 sporadic OCD cases and their unaffected parents to identify rare *de novo* (DN) single-nucleotide variants (SNVs). The primary aim of this pilot study was to determine whether DN variation contributes to OCD risk. To this aim, we evaluated whether there is an elevated rate of DN mutations in OCD, which would justify this approach toward gene discovery in larger studies of the disorder. Furthermore, to explore functional molecular correlations among genes with nonsynonymous DN SNVs in OCD probands, a protein–protein interaction (PPI) network was generated based on databases of direct molecular interactions. We applied Degree-Aware Disease Gene Prioritization (DADA) to rank the PPI network genes based on their relatedness to a set of OCD candidate genes from two OCD genome-wide association studies (Stewart *et al.*, 2013; Mattheisen *et al.*, 2014). In addition, we performed a pathway analysis with genes from the PPI network. The rate of DN SNVs in OCD was 2.51 × 10^−8^ per base per generation, significantly higher than a previous estimated rate in unaffected subjects using the same sequencing platform and analytic pipeline. Several genes harboring DN SNVs in OCD were highly interconnected in the PPI network and ranked high in the DADA analysis. Nearly all the DN SNVs in this study are in genes expressed in the human brain, and a pathway analysis revealed enrichment in immunological and central nervous system functioning and development. The results of this pilot study indicate that further investigation of DN variation in larger OCD cohorts is warranted to identify specific risk genes and to confirm our preliminary finding with regard to PPI network enrichment for particular biological pathways and functions.

## Introduction

The etiology of common neuropsychiatric disorders is believed to be multifactorial, with contributions from both environmental and genetic factors.^1^ Studies have predominantly focused on common polymorphic variants, finding small effect sizes, and replication of positive findings has been difficult.^2^ This raises the question of where the ‘missing heritability' of complex diseases might be found. A substantial portion may indeed reflect gene–gene interactions and gene–environmental interactions that have not been taken into consideration in estimates of narrow sense heritability.^3^ In addition, understanding the heritability of genetic diseases requires a more comprehensive assessment of human genetic variation, including rare variation, throughout the genome.^3, 4^

The emergence of next-generation sequencing platforms is facilitating comprehensive searches for both rare and common single-nucleotide variants (SNVs) across all genes in the genome via whole-exome sequencing (WES).^5^ Although protein-coding genes constitute only about 1% of the human genome, they are estimated to harbor 85% of mutations with large effects in disease-related traits.^6^

Several groups have identified genes conferring risk for intellectual disability,^7, 8^ schizophrenia^9, 10^ and autism^11, 12, 13, 14^ by applying WES in families with no previous history of these disorders or their related phenotypes—so-called sporadic or simplex families—and identifying recurrent *de novo* (DN) SNVs. The DN SNVs tend to be more common in patients than in controls or unaffected siblings, mainly when such variations are nonsynonymous and located in brain-expressed genes.^10, 11, 15^

Obsessive-compulsive disorder (OCD) is a severe neuropsychiatric disorder, commonly having an early age of onset, and is characterized by the presence of obsessions (unwanted, intrusive thoughts) and compulsions (repetitive behaviors) that can become incapacitating.^16, 17^ Family, twin, segregation and linkage studies suggest a complex genetic etiology, complicating the confirmation of specific risk variants; consequently, the cellular and molecular mechanisms underlying OCD pathophysiology remain uncertain.^18^

A meta-analysis of common variant genetic association studies of OCD found multiple polymorphisms with significant association.^19^ Significant variants were located in serotonergic genes, and, in males only, catecholamine modulation genes.^19^ The first published genome-wide association study (GWAS), querying common polymorphisms in a large cohort of OCD patients, did not find any variants reaching the genome-wide significance threshold, but top signals in several common variants were related to transcriptional regulation, cytoskeleton dynamics, ion channel assembly and gating, protein ubiquitination and degradation, and glutamate signaling.^20^ The latest GWAS in OCD^21^ also found no variants reaching genome-wide significance but showed significant overlap with top signals from the first GWAS. Furthermore, network analysis of top signals in these GWAS studies support the idea that genetic risk for OCD may cluster in certain biological networks or systems.^22^

There have been few studies examining rare variants in OCD.^23, 24, 25, 26, 27, 28, 29, 30, 31^ The first genome-wide investigation of rare copy number variation (CNV) in OCD and Tourette syndrome reported a 3.3-fold increase in large (>500 kb) deletions that overlap with CNVs reported in other neurodevelopmental disorders. An overall DN CNV rate of 1.4% was reported in OCD, slightly higher than the estimated frequency for controls, suggesting that rare DN variation may have a role in OCD pathogenesis.^32^ To date, there have been no published studies examining rare coding SNVs across the genome in OCD.

In the current pilot study, we examined 20 simplex OCD parent–child trios using WES to detect SNVs. Given the recent success in other neuropsychiatric disorders, we focused on detection of DN SNVs in OCD, aiming to advance our understanding of the contribution of rare nonsynonymous DN SNVs to the disorder. Next, we mapped these SNVs onto a protein–protein interaction (PPI) network, hypothesizing that perturbations of integrated molecular networks through genomic and environmental influences can increase the risk for complex diseases such as OCD^33^ and that topological properties of PPI networks represent molecular functional correlations among genes that are important for understanding disease biology. We then performed Degree-Aware Disease Gene Prioritization (DADA), ranking our SNVs against ‘seed' genes identified from two OCD GWAS, predicting that top-ranked genes in this analysis would achieve greater connectivity in our PPI network. Finally, we asked whether all the genes in our PPI network were enriched in certain canonical biological pathways, in an attempt to enhance our understanding of OCD pathophysiology.

## Materials and methods

### Subjects

This study was approved by the Research Ethics Committees of the University of São Paulo School of Medicine, as well as by the Brazilian National Commission of Research Ethics (CONEP, process number: 16756). All the participating subjects gave written informed consent.

The OCD patients, meeting DSM-IV criteria for the diagnosis, and their unaffected parents, were recruited at the Outpatient Clinic of the Obsessive-Compulsive Spectrum Disorders Program of the Institute of Psychiatry, at the University of São Paulo School of Medicine Hospital das Clínicas. The probands were evaluated by semi-structured and structured interviews included in the Brazilian Research Consortium on Obsessive-Compulsive Spectrum Disorders instruments, administered by trained clinicians (Supplementary Methods).^34^ The parents were directly screened with the Structured Clinical Interview for DSM-IV Axis I Disorders; those with any Axis I psychiatric diagnosis were excluded.

### Capture and sequencing

Exome capture, sequencing and variant detection were performed at the Yale Center for Genomic Analysis, as described previously^11^ and summarized below.

The DNA samples from whole blood were enriched for exonic sequence with the NimbleGen SeqCap EZ Exome v2 capture library (Roche NimbleGen, Madison, WI, USA). The samples were sequenced using the Illumina HiSeq 2000 platform (74 bp paired-end reads; Illumina, San Diego, CA, USA). We multiplexed four samples during each capture reaction and sequencing lane, pooling parents and probands when possible.

### Sequence alignment and variant calling

Short-read sequences were aligned to the human reference genome (hg19/NCBI 37) using the Burrows–Wheeler Aligner (http://bio-bwa.sourceforge.net/).^35^ The aligned reads were trimmed to the exome target using an in-house script. The trimmed and aligned data were converted to a sorted binary format, and duplicates were removed using SAMtools (http://samtools.sourceforge.net/),^36^ which was also used to identify the SNVs. SAMtools was selected for initial variant calling in order to replicate the bioinformatic pipeline used to analyze control subjects in the literature, which served as our comparison cohort for the overall rate of DN SNVs.^11^ For subsequent analyses of PPI networks, pathways and disease gene prioritization, we also considered confirmed variants from a second alignment and variant calling pipeline which followed the GATK v3 best practices guidelines (Supplementary Methods). The purpose of adding variants from this second pipeline was to ensure discovery of the maximum number of DN variants for our downstream analyses. The variants from both pipelines were annotated against the UCSC gene definitions (http://genome.ucsc.edu/) for impact on the encoded protein (silent, missense, nonsense and splice site) and for population allele frequency using ExAC v0.3.^37^

### Family relatedness check

A panel of informative genotypes was used to perform identity-by-descent in each study subject using the PLINK software package (http://pngu.mgh.harvard.edu/~purcell/plink/).^38^ Families were discarded if expected relationships did not confirm.

### DN SNVs

SNVs were predicted to be DN if all the following criteria were met: (1) the variant was not predicted in either parent, (2) at least eight unique reads supported the variant in the proband and (3) the locus had ⩾20 × coverage in both parents and the proband. We validated all the DN SNVs by PCR and Sanger sequencing (Supplementary Methods). We calculated the per-base rate of DN SNVs in our OCD samples and compared with rates reported in several published studies of reference populations and psychiatric disorders using a Poisson test (Supplementary Methods).

### PPI network analysis

Next, we mapped interactions between genes harboring validated nonsynonymous (missense or nonsense) DN SNVs by constructing a PPI network. A network is a set of elements interacting with each other through pairwise interactions. In the case of biological PPI networks, the components are the gene proteins (nodes) that are connected to each other by links (edges) representing known physical interactions between two components. We used Cytoscape^39^ and the iRefScape plugin^40^ to create a PPI network. This network is based on databases of direct physical interactions between genes, with proteins as nodes and interactions as undirected edges.^41^ We included PPIs that were present in any of the 10 databases consolidated within iRefIndex^40^ (Supplementary Methods). We applied two methods of permutation, ‘seed randomization' and ‘network permutation,' using GeneNet toolbox for MATLAB^42^ to show that the connectivity metrics of our network are different from networks generated by chance.

### DADA analysis

To investigate the potential relevance of our DN SNVs in OCD, we applied Degree-Aware Disease Gene Prioritization (DADA; http://compbio.case.edu/dada/), a tool for performing network-based prioritization of candidate disease genes.^43^ DADA analysis of our candidate genes harboring validated DN SNVs detected by WES were performed with the ‘seed' genes (curated from other studies as likely to be associated with the disorder) from two OCD GWAS reported to date^20, 21^ (Supplementary Methods). Prioritization is made based on the proximity of interaction (protein–protein interaction) of these seed genes with our WES candidate genes, using the NCBI Entrez Gene Database, which integrates human protein–protein interaction network data from several other databases such as HPRD, BioGRID and BIND.^44^

### Pathway analysis

To determine whether the list of all genes identified in our PPI network showed enrichment for specific biological pathways, we used Ingenuity Pathway Analysis (build version 355958M, content version 24718999; Ingenuity Systems, http://www.ingenuity.com/) to map genes to canonical pathways (Supplementary Methods). We also examined whether our confirmed DN missense variants in OCD were enriched for published lists of genes believed to contribute risk for autism, schizophrenia and intellectual disability^45^ using DNENRICH.^46^ Finally, we asked whether our PPI network gene lists showed enrichment for these disorders, calculating *P*-values in R (function phyper) based on a hypergeometric distribution.

**Figure 1:**
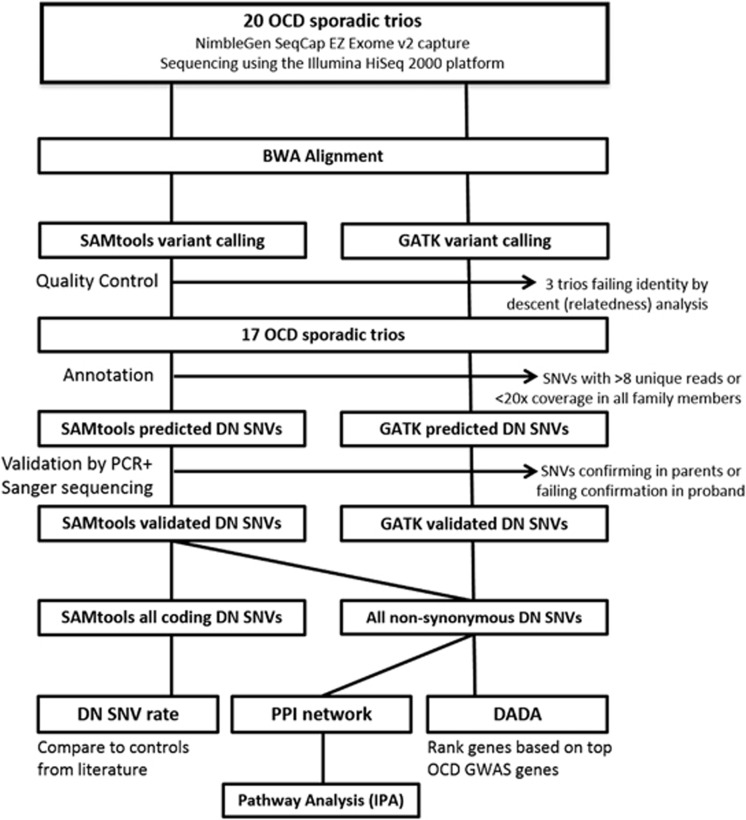
Single-nucleotide variant (SNV) discovery, quality control, annotation and analysis workflow. Whole-blood samples from obsessive-compulsive disorder (OCD) probands and their unaffected parents were enriched for exonic sequence with the NimbleGen SeqCap EZ Exome capture reagents and sequenced using the Illumina HiSeq 2000 platform. Identity by descent analysis was performed to confirm relatedness among samples. Final analyses included 17 OCD trios. Only *de novo* (DN) SNVs called by SAMtools and validated by Sanger sequencing (present in proband and absent in parents) were carried into DN SNV rate analyses. For subsequent analyses of protein–protein interaction (PPI), Degree-Aware Disease Gene Prioritization (DADA) and Ingenuity Pathway Analyses (IPA), we also included confirmed DN SNVs from a second alignment and variant calling pipeline, which followed the GATK v3 best practices guidelines.

A summary of all the methods and analyses is shown in Figure 1.

## Results

### Subjects

Our final analysis included WES data from 17 parent–child trios, each trio consisting of a child with OCD and their unaffected parents. Although we started with 20 trios, three were omitted from the analysis due to failing the family relatedness check. Among the 17 OCD trios included in our analyses, the mean age at symptom onset was 8.6 (±2.7) years and the mean Y-BOCS score was 26.1 (±5.7). Other clinical and demographic characteristics of included subjects are shown in Supplementary Table 1.

### Sequencing

On average, 91.6% of the generated sequence aligned to the reference genome, and 96.7% of the targeted bases in each individual were assessed by ⩾8 independent sequence reads. Only those bases with ⩾20 × coverage in all family members were considered for DN SNV detection, allowing for analysis of *de novo* events in 92.8% of all targeted bases (Supplementary Table 2).

### DN SNVs

Using the SAMtools variant detection pipeline (for comparison of DN SNV rate in OCD versus controls reported in the literature), 19 DN SNVs (12 nonsynonymous, 7 silent) were confirmed by Sanger sequencing (Table 1, Figure 1). Among nonsynonymous DN SNVs, we confirmed one nonsense mutation and 11 missense mutations. Of the 17 patients, 8 (~47%) carried at least one nonsynonymous DN SNV.

The total number of coding base pairs screened at ⩾20 × was 756.8 Mbp (Supplementary Table 3). Nineteen DN SNVs were validated, corresponding to a rate of 2.51 × 10^−8^ per base pair for human haploid genome and 1.12 events per trio. A two-tailed Poisson rate ratio test (R package rateratio.test) indicated that the rate of DN SNVs observed in our study differs significantly from that observed in unaffected siblings of autism probands, sequenced on the same platform and analyzed with the same bioinformatics pipeline^11^ (*P*=0.02, Table 2). There was no significant difference in paternal ages at conception between our OCD (mean 30.2 years) and this control cohort (mean 32.2 years; *P*=0.22, two-tailed Mann–Whitney test; Supplementary Table 4). We did not observe a significant difference when comparing our OCD DN SNV rate with those previously reported in schizophrenia,^10, 46^ autism^11, 13, 47, 48^ and intellectual disability^7, 8^ studies (Table 2).

Using the GATK v3 best practices variant calling pipeline, we confirmed eight additional nonsynonymous (all missense) DN SNVs, which we included in downstream analyses, for a total of 20 nonsynonymous DN SNVs (Table 1, Figure 1). There were no genes that harbored more than one confirmed DN SNV among the OCD subjects.

### PPI network and DADA analyses

**Figure 2:**
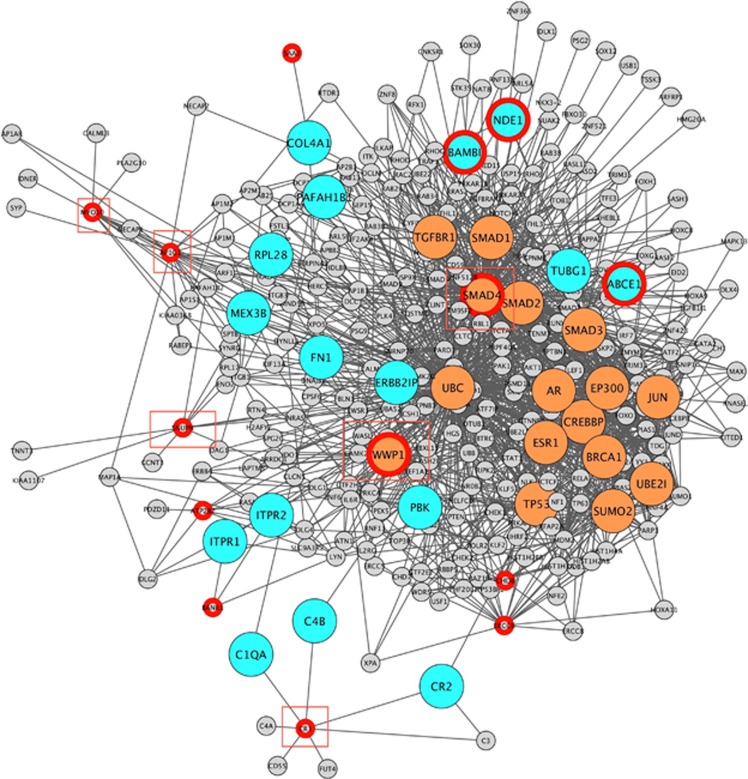
Protein–protein interaction network including nonsynonymous DN SNVs in OCD. Genes connecting components of the protein–protein interaction network harbor DN SNVs among the obsessive-compulsive disorder probands (red circles). Genes shaded blue are bridges, linking well-connected regions of the PPI network. Genes shaded orange are brokers, having a large number of connections with non-neighboring genes. Genes within red squares are bottlenecks, connecting different parts of the network with high betweenness. DN SNV, *de novo* single-nucleotide variant; OCD, obsessive-compulsive disorder; PPI, protein–protein interaction.

To construct the PPI network, we selected the genes with nonsynonymous (missense, nonsense) DN SNVs detected by our GATK and SAMtools pipelines (Figure 1, Table 1). Of the 20 genes harboring confirmed nonsynonymous DN SNVs, six genes (*FAM5B, CCDC108, VCX2*, *MUC5B*, *ARHGAP6*, *SLC35G5*) were not present in the PPI databases. Therefore, 14 genes served as the input for construction of our PPI network (Table 1). In the resulting PPI network of 320 nodes, two genes from our original inputs (*WWP1, SMAD4*) were found to be highly and independently interconnected with other non-neighboring genes (that is, they were found to be ‘brokers'), displaying 40 and 187 interactions (edges), respectively; six genes were found to be ‘bottleneck' genes (*WWP1, SMAD4, CR1, AP1G1, MYO10, SNUPN*) that connect different complexes or pathways in the network; and three were found to be ‘bridge' genes (*BAMBI*, *ABCE1, NDE1*) with high information flow, located between highly connected modules. (Figure 2, Supplementary Figure 1, Supplementary Table 5). Network permutations showed that connectivity metrics of ‘seed indirect degrees' mean and ‘common connectors means' for our network are different from these metrics in the permuted networks (1000 permutations).

Next, we performed DADA analysis to rank our 14 nonsynonymous DN SNVs in OCD subjects, using top genes from two OCD GWAS^20, 21^ as seed genes (Supplementary Table 6) to rank our DN SNVs. Genes with the highest prioritization rank using seeds from the first GWAS^20, 21^ were *BAMBI*, followed by *SMAD4* and *WWP1*. Using seed genes from the second GWAS,^20, 21^ highest prioritization rank was seen for *SMAD4*, followed by *WWP1* and *MYO10*. Of these four genes ranked highly by DADA, two are considered broker genes (*WWP1, SMAD4*) in our PPI network, three are considered bottleneck genes (*WWP1, SMAD4, BAMBI, MYO10*), and one is considered a bridge gene (*BAMBI*; Supplementary Table 5).

### Pathway analyses

Finally, a pathway analysis was performed to determine whether the nodes in our PPI network are enriched in canonical pathways from the Ingenuity Pathway Analysis database. We performed two pathway analyses using different gene lists as input: (1) using all 320 nodes in the PPI network (Supplementary Table 5) and (2) using 37 nodes from the PPI network determined to be brokers, bridges or bottlenecks by measurements of topological centrality (Supplementary Table 5), as these nodes may carry greater functional significance.^49, 50^ The analysis of all PPI network nodes found significant enrichment for pathways related to transforming growth factor beta (TGF-β) signaling, bone morphogenic protein signaling and glucocorticoid receptor signaling (Table 3). Narrowing the input list to bridges, brokers and bottlenecks also yields enrichment in TGF-β and glucocorticoid receptor signaling. Functional network enrichment for these central PPI nodes includes embryonic development, cell-to-cell signaling, cell death and survival, and cellular function and maintenance (Supplementary Table 9). There was no significant overlap between these same gene lists and lists of risk genes for autism, schizophrenia and intellectual disability^45^ (Supplementary Table 10).

## Discussion

To our knowledge, this is the first reported WES study in OCD designed to search for rare DN SNVs across all the coding regions of the genome in parent–child trios. As with similar studies in other neuropsychiatric disorders, the DN SNVs we identified may include true OCD risk variants and point toward relevant gene networks and canonical pathways. The small number of subjects in the present study precludes confirmation of the involvement of specific genes or variants in OCD. Nevertheless, the finding that DN SNVs occur more frequently in OCD is an essential prelude to identifying specific risk genes through the identification of recurrent mutations (independent DN variants mapping within the identical gene or at the same chromosomal locus) in larger patient cohorts. Furthermore, the network analyses in this pilot study integrate prior findings from GWAS^20, 21^ to generate hypotheses of potentially relevant genes, biological pathways, networks and processes in OCD that can be tested in larger studies.

Our first analysis used a SAMtools variant detection pipeline to compare the rate of confirmed DN SNVs in our OCD samples versus published rates in controls and other disorders. Our observed per base pair per generation DN SNV rate (2.51 × 10^−8^) differs significantly from a rate previously reported in unaffected subjects (1.31 × 10^−8^; ref. 11) using an identical variant calling pipeline. Parental age does not seem to account for this difference, and we did not find any difference between our rate and those reported in schizophrenia,^10, 46^ autism^11, 13, 47, 48^ or intellectual disability^7, 8^ exome-sequencing studies (Table 2).

In addition to the variants conformed using SAMtools, we confirmed eight additional nonsynonymous DN SNVs, predicted using a GATK best practices pipeline. We used all 20 nonsynonymous DN SNVs detected by both pipelines for downstream analyses. Given that protein gene products associated with disease have a higher likelihood of physically interacting,^41, 51, 52^ we next constructed a PPI network starting with candidate genes harboring confirmed nonsynonymous DN SNVs in OCD. Two of our candidate genes were classified as ‘brokers' (that is, highly and independently connected with non-neighboring genes) in this analysis, suggesting that they may be more relevant for disease (Supplementary Table 5).^52^ *WWP1* (*WW domain containing E3 ubiquitin protein ligase 1*) inhibits transcriptional activity induced by TGF-β, a member of a highly pleiotropic cytokine family that maintains immune homeostasis, directs lymphocyte differentiation and orchestrates aspects of embryonic development including neuronal migration and synapse formation.^53, 54^ *SMAD4* (SMAD family member 4) codes for a signal transduction protein that is activated by TGF-β signaling during proliferation and differentiation of the central nervous system.^55^ Furthermore, some of our candidate genes were classified as ‘bottlenecks' (key connectors) in the PPI network (Supplementary Table 5), believed to be one of the most significant indicators of essentiality.^56^ Aside from *WWP1* and *SMAD4*, other bottleneck genes were *CR1* (*complement component (3b/4b) receptor 1 [Knops blood group]*), an important member of the family of regulators of complement activation and a crucial multifunctional mediator of innate immunity; *MYO10 (myosin X*), which may have a role in neurite outgrowth and axon guidance;^57^ and *AP1G1* (*adaptor-related protein complex 1, gamma 1 subunit*), important for the formation of clathrin-coated vesicles to transport ligand–receptor complexes from the plasma membrane or from the trans-Golgi network to lysosomes.^58^ Although *AP1G1* has not been associated with any psychiatric disorder, it is noteworthy that the first genome-wide investigation of large, rare CNVs in OCD found a *de novo* CNV encompassing *AP1GBP1,* a related gene that also acts on clathrin-coated vesicles and may have a role in endocytosis.^32^

To investigate the potential relevance of our PPI network to OCD, we conducted a DADA analysis to see whether prioritized genes would have more connectivity in our PPI network. In this analysis, using a seed gene list curated from the two GWAS in OCD,^20, 21^ two of the three highest ranked genes (*WWP1* and *SMAD4*) were found to be brokers in the PPI network (Supplementary Table 7). The highest ranked gene in the DADA analysis using GWAS I (Supplementary Table 7) was *BAMBI* (*bone morphogenetic protein and activin membrane-bound inhibitor*), coding for a pseudoreceptor that negatively modulates TGF-β.^59^ *BAMBI* is classified as a bridge gene in our PPI network and is among the genes regulated by top-ranking SNPs in the OCD GWAS I secondary analysis;^20^ its protein product has been found to be selectively and significantly enriched in white matter progenitor cells, and can modulate their differentiation in oligodendrocytes or astrocytes.^60^

Furthermore, it is notable that the top-ranked genes in the DADA analyses *(BAMBI, SMAD4, WWP1, MYO10, AP1G1, ATP2B2*) have negative Residual Variation Intolerance Scores,^61^ indicating that in large exome-sequencing databases, they are found to contain less common functional genetic variation relative to the genome-wide expectation. Genes with negative Residual Variation Intolerance Scores are referred to as relatively ‘intolerant' to genetic variation and more likely to be involved with neurodevelopmental disease^61^ (Table 1, Supplementary Tables 7 and 8).

The canonical pathway analysis results from all the 320 nodes in our PPI network show enrichment for TGF-β signaling, bone morphogenic protein signaling and glucocorticoid signaling (Table 3); narrowing this list to 37 nodes classified as bridges, brokers and bottlenecks (Supplementary Table 5) shows enrichment for TGF-β and glucocorticoid receptor signaling. TGF-β signaling is mediated by a family of structurally related cytokines and by SMAD proteins that act to control proliferation, differentiation, migration and apoptosis of many different cell types. Bone morphogenic proteins are members of the TGF-β protein family of extracellular ligands,^62^ important in the neuronal protection against both apoptosis and excitotoxicity.^63^ The fidelity of these pathways are crucial for normal nervous system development and their disruption has been suggested to underlie schizophrenia pathology.^63^ Finally, glucocorticoids, a major subclass of steroid hormones, regulate a large number of immune, metabolic, cardiovascular and behavioral functions. Their major effects are anti-inflammatory via transcription induction of anti-inflammatory genes and by repression of inflammatory genes. Furthermore, their anti-inflammatory actions are complemented by their ability to induce apopotosis of cells, including monocytes and T lymphocytes. Imbalances in this pathway have been suggested in OCD, anxiety and other neuropsychiatric disorders.^64, 65, 66^

The major limitation of the current study is the small sample size. When studying rare variants, larger samples are needed to adequately show that certain genes or variants are associated with the disorder. Therefore, our study should be considered preliminary, requiring replication in larger OCD cohorts. Recruitment for a larger investigation of DN exome sequence variation in OCD trios is currently underway.

Although we are unable to pinpoint definitive risk genes or variants in a study of this size, we were able to show, for the first time, that (1) sporadic OCD families may have higher rates of DN coding SNVs, suggesting that further study of this type of variation in larger cohorts holds potential to identify risk genes; (2) a PPI network can be constructed on the basis of DN SNVs that appears to have relevance to OCD, judged against common variant findings from other genetic studies of OCD; and (3) PPI network genes show enrichment for biological pathways and functions involved with neurodevelopmental and immunological processes. These findings hold great promise for elucidating our understanding of OCD neurobiology and potential treatments, and deserve further scrutiny in larger cohorts.

## Figures and Tables

**Table 1:** Summary of confirmed *de novo* SNVs from exome sequencing in 17 OCD parent trios

*Individual*	*Chr*	*Position (hg19)*	*Gene*	*Expression*		*Reference allele*	*Variant allele*	*Variant effect*	*Amino acid substitution*	*RVIS (%ile)*	*ExAC frequency*	*Detection pipeline*
				*Brain*	*Synaptic*							
OCD016301	15	75891017	*SNUPN*	Yes	No	A	C	Missense	D255E	0.6 (82.66%)	NA	Both
OCD018901	3	10380029	*ATP2B2*	Yes	Yes	A	G	Missense	I1084T	−1.94 (1.89%)	NA	Both
OCD018901	3	180703745	*DNAJC19*	Yes	Yes	T	C	Silent	R83R	−0.08 (47.79%)	NA	SAMtools
OCD032201	2	219647088	*CYP27A1*	Yes	No	A	T	Silent	P61P	−0.31 (32.23%)	NA	SAMtools
OCD032201	8	87423972	*WWP1*	Yes	Yes	A	G	Missense	I310M	−0.69 (15.12%)	8.2 × 10^−^^6^	Both
OCD032201	10	50678378	*ERCC6*	Yes	No	T	A	Nonsense	K1210X	1.49 (95.32%)	NA	Both
OCD032201	11	62400166	*GANAB*	Yes	No	C	T	Silent	L311L	−0.66 (16.02%)	NA	SAMtools
OCD129101	2	219894325	*CCDC108*	Yes	No	C	T	Missense	E484K	−1.85 (2.04%)	8.2 × 10^−6^	Both
OCD129101	4	102993556	*BANK1*	Yes	No	A	T	Missense	K633M	0.45 (78%)	NA	Both
OCD129101	5	16783451	*MYO10*	Yes	No	C	T	Missense	E199K	−1.61 (2.97%)	NA	Both
OCD139801	1	89655829	*GBP4*	Yes	No	C	A	Silent	T363T	1.76 (96.73%)	NA	SAMtools
OCD139801	1	177226291	*FAM5B*	Yes	No	C	G	Missense	NA	−1.15 (6.27%)	NA	Both
OCD139801	1	207755290	*CR1*	No	No	T	A	Missense	S1748R	NA	NA	Both
OCD139801	2	223423326	*SGPP2*	Yes	No	C	T	Silent	P303P	−0.6 (17.91%)	1.7 × 10^−5^	SAMtools
OCD144601	2	220402768	*ACCN4*	Yes	No	G	A	Silent	X667X	0.4 (76.45%)	1.1 × 10^−5^	SAMtools
OCD144601	X	8138284	*VCX2*	Yes	No	G	C	Missense	A70G	NA	0.793	Both
OCD175901	16	11016048	*CIITA*	Yes	No	C	T	Silent	D1058D	−0.89 (10.19%)	4.1 × 10^−5^	SAMtools
OCD175901	16	71807129	*AP1G1*	Yes	Yes	T	C	Missense	K155E	−0.76 (13.45%)	NA	Both
OCD181401	10	28971325	*BAMBI*	Yes	No	G	A	Missense	V260I	−0.12 (45.13%)	8.24 × 10^−6^	Both
OCD176501	16	15790756	*NDE1*	Yes	No	A	C	Missense	A986C	−0.82 (11.77%)	0.0003	GATK
OCD018901	18	48584826	*SMAD4*	Yes	No	T	C	Missense	W302R	−0.32 (31.69%)	NA	GATK
OCD020001	11	1270892	*MUC5B*	Yes	No	C	A	Missense	A4261E	16.52 (99.98%)	0.001	GATK
OCD003301	X	11206984	*ARHGAP6*	Yes	No	G	A	Missense	S134F	−0.56 (19.73%)	0.008	GATK
OCD043301	14	21870199	*CHD8*	Yes	No	C	T	Missense	E1327K	−2.34 (1.18%)	NA	GATK
OCD048501	4	146033407	*ABCE1*	Yes	No	C	G	Missense	P243A	−0.25 (35.42%)	0.003	GATK
OCD048501	8	11188956	*SLC35G5*	Yes	Yes	G	A	Missense	S114N	1.36 (94.44%)	8.2 × 10^−6^	GATK
OCD048501	11	18266989	*SAA2*	Yes	No	T	C	Missense	K102E	0.73 (85.98%)	9.9 × 10^−5^	GATK

Abbreviations: Chr, chromosome; ExAC Frequency, overall variant frequencies in version 0.3 of the Exome Aggregation Consortium data set; NA, not present in data set; OCD, obsessive-compulsive disorder; RVIS, Residual Variation Intolerance Score, release 9 (http://genetic-intolerance.org, accessed December 1, 2015); SNV, single-nucleotide variant.

**Table 2:** *De novo* mutation rate comparisons between our OCD cohort and samples of affected and unaffected individuals evaluated in previous exome-sequencing studies

*Study*	*Observed rate*	*Poisson*, P	*95% Confidence interval*	*Phenotype*
Sanders *et al.*^10^	1.31 × 10^−8^	0.02	1.1–3.1	Unaffected sibling of autism proband
Sanders *et al.*^10^	1.58 × 10^−8^	0.89	0.93–2.57	Sporadic autism
Iossifov *et al.*^48^	2.00 × 10^−8^	0.40	0.75–1.99	Sporadic autism
O'Roak *et al.*^12^	2.17 × 10^−8^	0.60	0.68–1.84	Sporadic autism
Rauch *et al.*^7^	1.86 × 10^−8^	0.29	0.78–2.23	Intellectual disability
Girard *et al.*^9^	2.59 × 10^−8^	1	0.47–2.05	Schizophrenia
Xu *et al.*^8^	1.73 × 10^−8^	0.17	0.85–2.34	Schizophrenia
Fromer *et al.*^46^	1.61 × 10^−8^	0.09	0.93–2.46	Schizophrenia

Abbreviation: OCD, obsessive-compulsive disorder.

Observed rates are *de novo* variants observed per base pair per generation in the sequenced exome; statistical comparison is between the observed rate and our OCD cohort rate of 2.51 × 10^-8^ using a two-tailed exact rate ratio test of two Poisson counts; 95% confidence interval is the observed rate/OCD cohort rate.

**Table 3:** IPA canonical pathway enrichment analysis of PPI network nodes

*IPA pathway*	P-value	*Overlap*	*Genes*
TGF-β signaling	1.3E−25	26/87	*TGFBR1, MAPK1, SMAD3, SKI, SMAD5, MAPK13, TGIF1, EP300, TGFBR2, JUN, RUNX2, SMAD4, NF4A, TFE3, SMAD1, SMAD2, NRAS, SMAD9, FOXH1, CREBBP, SMAD6, SSMAD7, ZNF423, IRF7, RRAS2, PIAS4*

BMP signaling pathway	2.5E−15	18/74	*RELA, NRAS, SMAD9, MAPK1, CREBBP, SMAD6, SMAD7, PRKAR2A, MAPK13, SMAD5, ATF2, ZNF423, RRAS2, JUN, RUNX2, PRKAR1B, SMAD4, SMAD1*

Glucocorticoid receptor signaling	7.1E−12	25/272	*RELA, SMAD2, TGFBR1, NRAS, MAPK1, SMAD3, CREBBP, GTF2E2, MAPK13, CEBPB, EP300, TGFBR2, JUN, AKT1, POLR2A, RRAS2, AR, SUMO1, FOXO3, PRKAA2, GTF2H1, SMAD4, STAT1, ESR1, UBE2I*

Abbreviations: BMP, bone morphogenic protein; IPA, Ingenuity Pathway Analysis; PPI, protein–protein interaction; TGF-β transforming growth factor beta.

The IPA database contained 320 gene nodes from the PPI network, built from nonsynonymous *de novo* single-nucleotide variants in obsessive-compulsive disorder probands. The top pathways with the lowest *P*-values are shown.
