# Supplementary material for: Whole-exome sequencing in obsessive-compulsive disorder identifies rare mutations in immunological and neurodevelopmental pathways
Source: Transl Psychiatry. 2016 Mar 29;6(3):e764–. doi: 10.1038/tp.2016.30 (PMC4872454; doi:10.1038/tp.2016.30)
Supplement: Supplementary Information [file tp201630x1.docx]

**SUPPLEMENTARY METHODS**

Subjects

The inclusion criteria for OCD patients were: between 18 and 65 years of age; having a primary diagnosis of OCD, according to DSM-IV criteria, with a reported symptom onset before 16 years of age; and having a Yale-Brown Obsessive-Compulsive Scale (Y-BOCS)^1^ total score of at least 16 when both obsessions and compulsions were present, or at least 10 when only obsessions or compulsions were present.

Subjects with a primary diagnosis of a psychotic disorder or any other condition that could impair their understanding of the protocol questions were excluded. Patients with a clinical condition that could hinder interpretation of the results were excluded, including those with onset of OCD symptoms after significant head trauma or secondary to other neurological disorders.

All subjects were evaluated with semi-structured and structured interviews included in the Brazilian Research Consortium on Obsessive-Compulsive Spectrum Disorders instrument, administered by trained interviewers using the quality control protocols described elsewhere^2^.

Family histories were obtained using a questionnaire that addressed various psychiatric conditions, including phobias, anxiety, and depression, as well as treatment for any of these conditions.

Exome capture, sequencing, and variant detection overview

Whole blood samples were enriched for exonic DNA using the NimbleGen SeqCap EZ Exome v2 library (45Mbp target). Exon-enriched DNA was sequenced using the Illumina HiSeq 2000, running 4 samples per lane, generating 74 base pair paired-end reads. Capture and sequencing was performed at the Yale Center for Genomic Analysis (YCGA). Sequencing data were run through the CASAVA pipeline (Illumina) and then aligned to the entire human genome reference sequence (hg19/NCBI 37) with the Burrows-Wheeler Aligner (BWA). Aligned reads were trimmed to the exome target using an in-house script. If any read overlapped ≥1 bp of a probe in the NimbleGen capture library, the read was considered “on-target”. The reads that did not meet this criterion were discarded. The trimmed and aligned data were converted to a sorted binary format (BAM), and duplicates were removed with SAMtools, which was also used to identify SNVs. To remove systematic errors from the Illumina data, the variants were assessed using SysCall^3^. This algorithm was trained using discrepant variants detected in overlapping paired-end data and uses a combination of read-direction, quality scores and surrounding sequence to identify systematic errors.

Alignment and SAMtools conversion

Rescaled FASTQ format data were aligned to unmasked human genome build 19 (NCBI 37) using BWA with the default settings using the following command: bwa aln -t 8 ‘BWA_reference’ ‘Fastq_input’ > ‘Output.sai’.

Aligned reads were converted to SAMtools format using the following command: bwa sampe ‘BWA_reference’ ‘Output_pair1.sai’ ‘Output_pair2.sai’ ‘Fastq_input_pair1’ ‘Fastq_input_pair2’ > ‘Output.sam’.

Duplicate removal and pileup conversion

The trimmed aligned data were converted to BAM format (sorted and aligned), and duplicates were removed using SAMtools with the default settings and the following commands: samtools view -bSt ‘SAM_reference’ ‘Input.sam’ | samtools sort – ‘Output.bam’, followed by: samtools rmdup -u ‘Input.bam’ - | samtools view - -o ‘Output.sam’.

The aligned, trimmed, and duplicate-free SAM file was then converted to pileup format using SAMtools with the default settings: samtools pileup -cAf ‘Reference’ -t ‘SAM_reference’ ‘Input.sam’ > ‘Output.pileup’.

Variant detection and data cleaning

Variants from the reference genome were filtered from the pileup file using the SAMtools variant filter script and the following command: samtools.pl varFilter -d 4 -D 10000000 ‘Input.pileup’ > ‘Output.var’.

To remove systematic errors from the Illumina data, the variants were assessed using SysCall. The algorithm was used with default settings and following command: SysCall.pl ‘Input.var’ ‘Input.sam’ ‘Output’ ‘Path’. All genome positions that were present in the ‘Error’ file in one or more samples were removed from the dataset.

GATK alignment and variant calling pipeline

For all comparisons between DN SNV rates in our OCD cases versus controls from the literature, variant detection using SAMtools was performed as detailed above. This was to ensure that the methods used to detect variants in our cases matched the methods used controls. To ensure discovery of the maximum number of DN variants for subsequent network, pathway, and systems analyses, we added variants called from a second pipeline, in which alignment and variant calling of the sequencing reads followed the GATK v3 best practices guidelines. Reads were aligned using BWA MEM (v. 0.7.10) to the b37 human reference sequence with decoy sequences. Picard's Mark Duplicates tool (v. 1.118) was used to mark PCR duplicates, then the GATK software (v. 3.2.3) was used to realign indels, and recalibrate quality scores. The analysis used GATK's best practice parameters, and the default parameters for BWA and Picard. A verification analysis, not shown, determined that the optimal variant call thresholds for exome data generated by the Yale Center for Genome Analysis matched the default best practice parameters. The target bed file was created by taking the union of the Nimblegen EZExome V2 probe regions, padded by 50 bases on each side, and the Nimblegen EZExome V2 target regions, padded by 40 bases on each side. This ensures that all of the regions expected to be captured in sufficient quality to call variants are included in the analysis.

*De novo* (DN) single nucleotide variant (SNVs) detection

Sequence data for each variant in a proband were compared with their parents at the same position. A variant was predicted to be DN if it was not predicted in either parent (single parent for chrX and chrY in male offspring). Data were normalized within families by analyzing only the bases with at least 20 unique reads in all family members. Additionally, we required at least 8 unique reads supporting the variant in the offspring, at least 90% of reads supporting the reference in both parents (single parent for chrX and chrY in male offspring), and a mean PHRED-like quality score of at least 15 for reads supporting the variant.

All DN SNV predictions were validated experimentally using PCR to amplify the region from whole-blood derived DNA in all family members. Sanger dideoxynucleotide sequencing was performed at the Yale Keck facility, http://medicine.yale.edu/keck) to confirm the variant was present only in the proband and in both the forward and reverse directions. Primers were designed using Primer3 and oligonucleotides were synthesized by Integrated DNA Technologies (IDT, http://www.idtdn.com).

The DN variants we observed and confirmed are single nucleotide variant (SNV) substitutions (e.g. A->T giving a genotype of AT) that are present in the child, but absent in both parents (AA and AA). The presence of this novel nucleotide will be unaffected by copy number in either the parents or the child, so we do not control for potential CNVs in our data. The presence of a heterozygous variant makes a deletion CNV implausible (it would show either an 'A' or a 'T', but an AT would not be possible). While it is possible that an overlapping duplication was present (giving a genotype of ATT or AAT) this would not alter the fact that a DN nucleotide substitution had occurred since the 'T' was not present in either parent. There is no reason to suspect that a duplication at this loci is any more likely than at any other location in the genome. Should a duplication be present, it would not alter the association described in this paper.

Quality control

As described in the main text, prompted by the prediction of an excessive number of *de novo* variants (>1,000) in three of our starting 20 OCD trios, we performed identity-by-descent analysis in all families, using the Plink software package (http://pngu.mgh.harvard.edu/~purcell/plink/) and 297 informative SNPs extracted from the exome data using a custom script. This analysis revealed non-paternity in these three OCD trios and expected family structure in the remaining trios. Consequently, these three trios were omitted from further analyses. Final analyses included 17 OCD parent-child trios.

Variant annotation

Variants were annotated against the UCSC gene definitions (http://genome.ucsc.edu/) to determine the effect on the resulting amino acid sequence. Where multiple isoforms were present, the most-deleterious interpretation was selected. Coding DN SNVs were also annotated for their frequency in over 60,000 exomes (ExAC v0.3)^4^ and genes were annotated for Residual Variation Intolerance Scores^5^ and for their expression in human brain. A list of brain-expressed genes was obtained from a study of the human brain transcriptome throughout development and adulthood^6^. Genes were annotated as ‘synaptic’ if they were implicated in prior proteomic analyses^7-9^.

Rate of DN variation

Using the total number of on-target sequenced reads and the estimates of sequence coverage, we calculated the total of bases analyzed (i.e., bases with at the least 8 reads supporting the variant, and with at least 20x coverage in all family members). As described in the main text, the number of DNMs found in each OCD proband was tabulated and the rate observed was calculated by dividing this number by the number of bases analyzed. We compared this DN mutation rate in OCD to rates reported in several published studies of reference populations and psychiatric disorders using a two-tailed Poisson rate ratio test (R package rateratio.test). The variances of the two groups differed with regard to number of DN mutations (OCD 2.1, controls 0.56, ratio of variances 3.78, Levene test for two variances p=0.02) and Poisson rate ratio test also differed (p=0.02, Table 2), indicating that the rate of DN SNVs observed in our study differs significantly from that observed in unaffected siblings of autism probands, sequenced on the same platform and analyzed with the same bioinformatics pipeline^10^. There was no significant difference in paternal ages at conception between our OCD (mean 30.2 years) and this control cohort (mean 32.2 years) (p=0.22, two-tailed Mann-Whitney test) (Table S4), and the variances in these two groups did not differ (OCD 26.4, controls 37.6, ratio of variances 0.7, Levene test for two variances p=0.68).

Protein-protein interaction (PPI) network analysis

As described in the main text, we examined interactions among genes harboring non-synonymous DN SNVs by constructing a PPI network based on known physical interactions among their protein products.^11^ We used Cytoscape^12^ and the iRefScape^13^ plugin, containing the iRefIndex database that consolidates protein interaction data from ten databases to create a PPI network.

We calculated measures of topological centrality, including degree, betweenness, clustering coefficient, and bridging of this PPI network^12, 14^. These topological centrality metrics were then used to identify “brokers” (nodes with high degree that connect many nodes that would not be connected otherwise), “bridges” (nodes with high information flow that are located between highly connected modules), and “bottlenecks” (nodes with the highest betweenness that connect different complexes or pathways in the network)^14, 15^. We used the 95th percentile as a distribution threshold to select the best brokers, bridges, and bottlenecks.

The betweenness of a node is based on the number of shortest paths that pass through a node *i*:


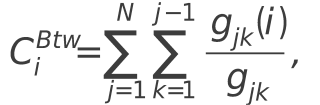


where g_jk_(i) is the number of shortest-paths from node *j* to node *k* passing through *i* and g_jk_ is the total of shortest-paths between *j* and *k*.

Reciprocally a node’s degree corresponds to its number of interaction partners and is a local measure of centrality.^16^

The clustering coefficient of a node is the ratio of the number of links between the neighbors of a node divided by the total connections that could exist among them:


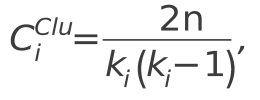


where *n* is the number of edges among the neighbors of node *I,* and k_i_ is the degree of node *i*. This measure varies between 0 and 1.

Bridging centrality measures the extent to which a node or an edge is located between well-connected regions: ^17^


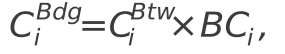


where C_i_^Btw^ is the betweenness centrality of node i, and BC_i_ is the coefficient that evaluates the local characteristics of the bridge in the neighborhood of node i, that is defined below:


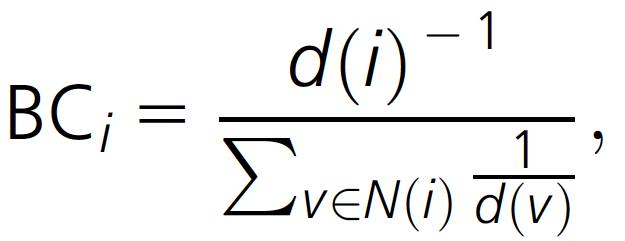


where *d(i)* is the degree of node *i* and *N(i)* is the set of neighbors of node *i*.

Based on the hypothesis that PPI network genes associated with complex and Mendelian diseases have an unusually high number of connections (high degree) with a low number of connections among their neighbors (low clustering coefficient)^14^, we looked for such “broker” genes in the network, using topological measures. We also looked for non-hub genes (low degree) that linked well-connected regions of the PPI network (“bridges”) and “bottlenecks” (high degree and high betweenness).

Because the PPI databases are built based on previously studied protein interactions, there is a bias toward representation of the most widely studied genes. Therefore, it is important to determine the significance of connectivity among the PPI network genes. To detect whether such bias affected our results, we used GeneNet Toolbox for MATLAB to perform a ‘network permutation’ method for calculating the significance of connectivity among seed-genes. This method holds seed-genes constant while permuting the edges of the network many times (preserving node degree and network clustering structure), and obtaining an empirical p-value by comparing seed connectivity (direct and indirect) in the original network versus random networks.^18, 19^

DADA - degree-aware disease gene prioritization analysis

To further investigate the relevance of our PPI network to OCD, we applied degree-aware disease gene prioritization analysis (DADA)^20^, which uses a degree-aware algorithm to rank candidate genes, taking into account a list of seed genes, curated as likely to be involved with OCD risk (Table S5). For this analysis, we used the following seed lists: (1) genes considered to be quantitative trait loci for the top 38 OCD-associated single nucleotide polymorphisms (SNPs) in an OCD GWAS^21^ with *P*-values < 5 × 10^−5^. These top SNPs were annotated with quantitative trait loci, expression (eQTLs) and methylation level (mQTL) data. In the seed list, we also included genes whose eQTLs or mQTLs are associated (*P*-value < 0.05) with these specified SNP^21^; (2) genes with SNPs listed as strongest associated GWAS variants (*P* < 0.0001) in the hybrid analysis of the within- and between-family component^22^; genes were included if they physically overlapped with the SNP, otherwise the nearest flanking gene was included. We performed DADA analyses using both GWAS gene lists together, then for each seed gene list separately.

For the OCD candidate gene set, we used genes found to harbor confirmed non-synonymous DN SNVs in the present study. We emphasize that this ranking does not imply causality in OCD but rather relatedness to genes previously and independently associated with OCD.

Pathway analyses

To determine whether the nodes in our PPI network (generated from non-synonymous DN SNVs) are enriched for specific biological pathways, we identified the most significant canonical pathways suggested by Ingenuity Pathway Analysis (IPA, build version 355958M, content version 24718999; Ingenuity Systems, http://www.ingenuity.com/). The following default settings were used: Reference set: Ingenuity Knowledge Base (Genes Only); direct and indirect relationships; does not include endogenous chemicals; consider only relationships where species = human and confidence = experimentally observed.

**References**

1. Goodman WK, Price LH, Rasmussen SA, Mazure C, Fleischmann RL, Hill CL *et al.* The Yale-Brown Obsessive Compulsive Scale. I. Development, use, and reliability. *Arch Gen Psychiatry* 1989; **46**(11)**:** 1006-1011.

2. Miguel EC, Ferrão YA, Rosário MC, Mathis MA, Torres AR, Fontenelle LF *et al.* The Brazilian Research Consortium on Obsessive-Compulsive Spectrum Disorders: recruitment, assessment instruments, methods for the development of multicenter collaborative studies and preliminary results. *Rev Bras Psiquiatr* 2008; **30**(3)**:** 185-196.

3. Meacham F, Boffelli D, Dhahbi J, Martin DI, Singer M, Pachter L. Identification and correction of systematic error in high-throughput sequence data. *BMC Bioinformatics* 2011; **12:** 451.

4. Lek M, Karczewski K, Minikel E, Samocha K, Banks E, Fennell T *et al.* Analysis of protein-coding genetic variation in 60,706 humans. *bioRxiv* 2015.

5. Petrovski S, Wang Q, Heinzen EL, Allen AS, Goldstein DB. Genic intolerance to functional variation and the interpretation of personal genomes. *PLoS Genet* 2013; **9**(8)**:** e1003709.

6. Naumova OY, Lee M, Rychkov SY, Vlasova NV, Grigorenko EL. Gene expression in the human brain: the current state of the study of specificity and spatiotemporal dynamics. *Child Dev* 2013; **84**(1)**:** 76-88.

7. Bayés A, van de Lagemaat LN, Collins MO, Croning MD, Whittle IR, Choudhary JS *et al.* Characterization of the proteome, diseases and evolution of the human postsynaptic density. *Nat Neurosci* 2011; **14**(1)**:** 19-21.

8. Collins MO, Husi H, Yu L, Brandon JM, Anderson CN, Blackstock WP *et al.* Molecular characterization and comparison of the components and multiprotein complexes in the postsynaptic proteome. *J Neurochem* 2006; **97 Suppl 1:** 16-23.

9. Abul-Husn NS, Bushlin I, Morón JA, Jenkins SL, Dolios G, Wang R *et al.* Systems approach to explore components and interactions in the presynapse. *Proteomics* 2009; **9**(12)**:** 3303-3315.

10. Sanders SJ, Murtha MT, Gupta AR, Murdoch JD, Raubeson MJ, Willsey AJ *et al.* De novo mutations revealed by whole-exome sequencing are strongly associated with autism. *Nature* 2012; **485**(7397)**:** 237-241.

11. Gandhi TK, Zhong J, Mathivanan S, Karthick L, Chandrika KN, Mohan SS *et al.* Analysis of the human protein interactome and comparison with yeast, worm and fly interaction datasets. *Nat Genet* 2006; **38**(3)**:** 285-293.

12. Saito R, Smoot ME, Ono K, Ruscheinski J, Wang PL, Lotia S *et al.* A travel guide to Cytoscape plugins. *Nat Methods* 2012; **9**(11)**:** 1069-1076.

13. Razick S, Mora A, Michalickova K, Boddie P, Donaldson IM. iRefScape. A Cytoscape plug-in for visualization and data mining of protein interaction data from iRefIndex. *BMC Bioinformatics* 2011; **12:** 388.

14. Cai JJ, Borenstein E, Petrov DA. Broker genes in human disease. *Genome Biol Evol* 2010; **2:** 815-825.

15. Yu H, Kim PM, Sprecher E, Trifonov V, Gerstein M. The importance of bottlenecks in protein networks: correlation with gene essentiality and expression dynamics. *PLoS Comput Biol* 2007; **3**(4)**:** e59.

16. Barabási AL, Oltvai ZN. Network biology: understanding the cell's functional organization. *Nat Rev Genet* 2004; **5**(2)**:** 101-113.

17. Doncheva NT, Assenov Y, Domingues FS, Albrecht M. Topological analysis and interactive visualization of biological networks and protein structures. *Nat Protoc* 2012; **7**(4)**:** 670-685.

18. Taylor A, Steinberg J, Andrews TS, Webber C. GeneNet Toolbox for MATLAB: a flexible platform for the analysis of gene connectivity in biological networks. *Bioinformatics* 2015; **31**(3)**:** 442-444.

19. Rossin EJ, Lage K, Raychaudhuri S, Xavier RJ, Tatar D, Benita Y *et al.* Proteins encoded in genomic regions associated with immune-mediated disease physically interact and suggest underlying biology. *PLoS Genet* 2011; **7**(1)**:** e1001273.

20. Erten S, Bebek G, Ewing RM, Koyutürk M. DADA: Degree-Aware Algorithms for Network-Based Disease Gene Prioritization. *BioData Min* 2011; **4:** 19.

21. Stewart SE, Yu D, Scharf JM, Neale BM, Fagerness JA, Mathews CA *et al.* Genome-wide association study of obsessive-compulsive disorder. *Mol Psychiatry* 2012.

22. Mattheisen M, Samuels JF, Wang Y, Greenberg BD, Fyer AJ, McCracken JT *et al.* Genome-wide association study in obsessive-compulsive disorder: results from the OCGAS. *Mol Psychiatry* 2014.
