# Supplementary material for: Whole-exome sequencing in obsessive-compulsive disorder identifies rare mutations in immunological and neurodevelopmental pathways
Source: Transl Psychiatry. 2016 Mar 29;6(3):e764–. doi: 10.1038/tp.2016.30 (PMC4872454; doi:10.1038/tp.2016.30)
Supplement: Supplementary Table [file tp201630x2.docx]

**Table S1.**

**Clinical and demographic characteristics of obsessive-compulsive disorder probands**

| *ID* | *Sex* | *Age at symptom onset* | *Y-BOCS* | *Medical history* | *Psychiatric comorbidities* |
| --- | --- | --- | --- | --- | --- |
|  |  | *(Years)* | *Total score* |  |  |
| OCD0033 | M | 7 | 37 | NA | Tourette, social phobia, self-injury, MDD, GAD, BDD, PTSD |
| OCD0163 | M | 11 | 22 | Migraine | Tics, social phobia, specific phobia, GAD, skin picking |
| OCD0187 | F | 12 | 24 | NA | MDD, social phobia, skin picking, panic with agoraphobia |
| OCD0189 | M | 8 | 30 | No | Dysthymic disorder |
| OCD0200 | M | 12 | 21 | NA | MDD |
| OCD0216 | F | 8 | 25 | NA | MDD, alcohol use disorder, skin picking, agoraphobia without panic, BDD, eating disorder |
| OCD0322 | F | 7 | 19 | NA | NA |
| OCD0477 | M | 5 | 28 | NA | GAD, MDD |
| OCD1291 | M | 14 | 28 | Cerebral palsy, hand tremor, asthma, bronchitis | Tourette, agoraphobia without panic, specific phobia, GAD |
| OCD1353 | F | 5 | 17 | NA | Specific phobia, BDD, skin picking, GAD, self injury |
| OCD1398 | F | 7 | 26 | Migraine | Tics, specific phobia, social phobia, BDD, dysthymic disorder |
| OCD1446 | M | 10 | 20 | NA | Tics, skin picking, MDD |
| OCD1759 | M | 10 | 30 | NA | MDD, ADHD, GAD |
| OCD1765 | F | 6 | 29 | NA | GAD, skin picking, ADHD |
| OCD1814 | M | 5 | 33 | Trauma | GAD, PTSD, MDD, ADHD |
| OCD0485 | M | 10 | 34 | NA | Social phobia, MDD |
| OCD0433 | M | 8 | 21 | NA | Tourette |

Y-BOCS, Yale-Brown Obsessive-Compulsive Scale; OCD, obsessive-compulsive disorder; MDD, major depressive disorder; GAD, generalized anxiety disorder; BDD, body dysmorphic disorder; PTSD, post-traumatic stress disorder; ADHD, attention deficit hyperactivity disorder.

**Table S2.**

**Overview of exome sequencing data for all 17 trios (n = 51 samples) passing quality control**

| *Variable* | *Mean (95% CI)* |
| --- | --- |
| Total reads (millions) | 107.0 (±8.1%) |
| % of aligned reads | 91.6% (±0.001%) |
| % of on-target reads | 66.4% (±0.003%) |
| % duplicate reads | 5.5% (±0.004%) |
| Mean coverage | 80x (±8.1x) |
| % of target at 4× | 97.8% (±0.001%) |
| % of target at 8× | 96.7% (±0.002%) |
| % of target at 20× | 92.8% (±0.008%) |
| Mean base pair error rate | 0.41% (±0.01%) |
| Transition/transversion ratio | 2.61 (±0.004%) |

**Table S3.**

**Coding sequence with at least 20x coverage for each proband and in total (756.8 Mbp)**

| Sample | Total Coding sequence  (Mbp) coverage 20x | Total DNMs |  |
| --- | --- | --- | --- |
| OCD175901 | 44.1 | 2 |  |
| OCD043301 | 46.6 | 0 |  |
| OCD047701 | 46.0 | 0 |  |
| OCD029101 | 45.2 | 3 |  |
| OCD135301 | 44.3 | 0 |  |
| OCD048501 | 37.6 | 0 |  |
| OCD139801 | 43.9 | 4 |  |
| OCD176501 | 44.7 | 0 |  |
| OCD144601 | 46.1 | 2 |  |
| OCD181401 | 45.3 | 1 |  |
| OCD003301 | 45.1 | 0 |  |
| OCD016301 | 42.3 | 1 |  |
| OCD018701 | 45.1 | 0 |  |
| OCD018901 | 45.8 | 2 |  |
| OCD020001 | 42.9 | 0 |  |
| OCD021601 | 45.7 | 0 |  |
| OCD032201 | 46.0 | 4 |  |
| Total: | 756.8 | 19 | = 2.51x10^-8^ per base pair per generation |

**Table S4.**

**Paternal ages at conception of OCD and control subjects**

See Excel file.

**Table S5.**

**Nodes and calculated measurements of topological centrality in a PPI network constructed from non-synonymous DN SNVs in OCD**

See Excel file

**Table S6.**

**Seed genes from OCD GWAS (Stewart et al., 2013; Mattheisen et al., 2014) used for Degree-Aware Disease Gene Prioritization Analysis (DADA)**

See Excel file

**Table S7.**

**DADA analysis using seed genes from OCD GWAS I**

14 genes/proteins with non-synonymous DN SNVs found in OCD trios and contained in the protein-protein interaction (PPI) network. Genes are ranked by degree-aware disease gene prioritization analysis (DADA), with summary attributes. Seed genes for DADA are from the first published OCD GWAS (Stewart et al., 2013).

| **Protein node** | **Rank** | **Degree** | **Clustering coefficient** | **Connected component?** | **Bridge, Broker or bottleneck gene?** |
| --- | --- | --- | --- | --- | --- |
| BAMBI | 1 | 5 | 0.40000 | Yes | Yes |
| SMAD4 | 2 | 187 | 0.04261 | Yes | Yes |
| WWP1 | 3 | 40 | 0.06923 | Yes | Yes |
| AP1G1 | 4 | 21 | 0.11429 | Yes | Yes |
| ATP2B2 | 5 | 8 | 0.28571 | Yes | No |
| ERCC6 | 6 | 22 | 0.21212 | Yes | No |
| MYO10 | 7 | 10 | 0.15556 | Yes | Yes |
| CHD8 | 8 | 11 | 0.36364 | Yes | No |
| NDE1 | 9 | 4 | 0.00000 | Yes | No |
| SNUPN | 10 | 11 | 0.12727 | Yes | Yes |
| CR1 | 11 | 7 | 0.04762 | Yes | Yes |
| ABCE1 | 12 | 4 | 0.16667 | Yes | No |
| BANK1 | 13 | 3 | 0.33333 | Yes | No |
| SAA2 | 14 | 1 | 0.00000 | Yes | No |
|  |  |  |  |  |  |

**Table S8.**

**DADA analysis using seed genes from OCD GWAS II**

14 genes/proteins with non-synonymous DN SNVs found in OCD trios and contained in the protein-protein interaction (PPI) network. Genes are ranked by degree-aware disease gene prioritization analysis (DADA), with summary attributes. Seed genes for DADA are from the second published OCD GWAS (Mattheisen et al., 2014).

| **Protein node** | **Rank** | **Degree** | **Clustering coefficient** | **Connected component?** | **Bridge, Broker or bottleneck gene?** |
| --- | --- | --- | --- | --- | --- |
| SMAD4 | 1 | 187 | 0.04261 | Yes | Yes |
| WWP1 | 2 | 40 | 0.06923 | Yes | Yes |
| MYO10 | 3 | 10 | 0.15556 | Yes | Yes |
| ATP2B2 | 4 | 8 | 0.28571 | Yes | No |
| ERCC6 | 5 | 22 | 0.21212 | Yes | No |
| AP1G1 | 6 | 21 | 0.11429 | Yes | Yes |
| CHD8 | 7 | 11 | 0.36364 | Yes | No |
| NDE1 | 8 | 4 | 0.00000 | Yes | No |
| SNUPN | 9 | 11 | 0.12727 | Yes | Yes |
| BAMBI | 10 | 5 | 0.40000 | Yes | Yes |
| ABCE1 | 11 | 4 | 0.16667 | Yes | No |
| BANK1 | 12 | 3 | 0.33333 | Yes | No |
| CR1 | 13 | 7 | 0.04762 | Yes | Yes |
| SAA2 | 14 | 1 | 0.00000 | Yes | No |
|  |  |  |  |  |  |

**Table S9. IPA top biological functions and networks of PPI network nodes**


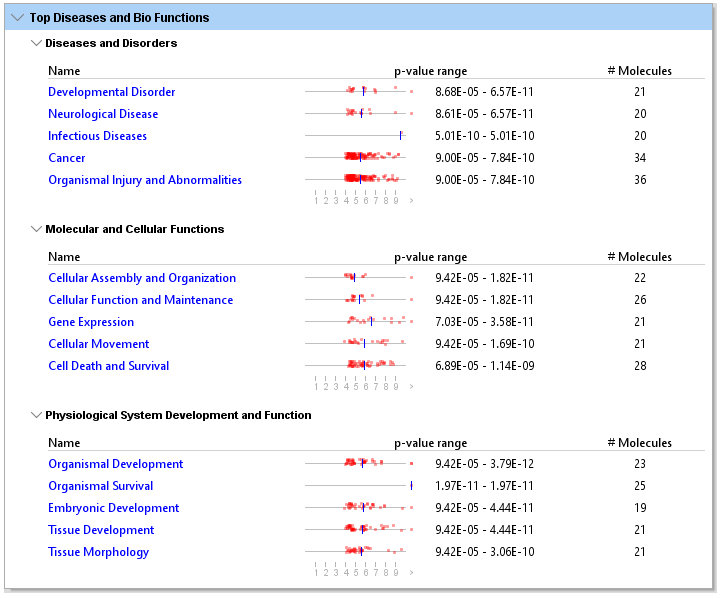

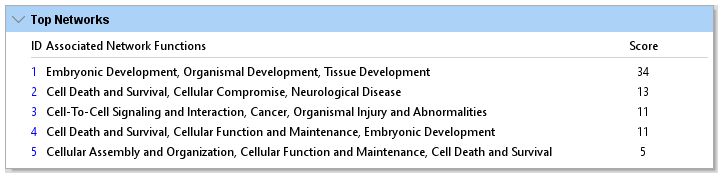


**Table S10. Overlap probabilities between PPI network nodes and gene lists for autism, schizophrenia, and intellectual disability**

See Excel workbook
