# Supplementary material for: Whole-exome sequencing in obsessive-compulsive disorder identifies rare mutations in immunological and neurodevelopmental pathways
Source: Transl Psychiatry. 2016 Mar 29;6(3):e764–. doi: 10.1038/tp.2016.30 (PMC4872454; doi:10.1038/tp.2016.30)
Supplement: Supplementary Figure Legend [file tp201630x3.docx]

**Figure S1.**

**Best brokers, bottlenecks and bridges in a protein-protein interaction (PPI) network.** We used the 95th percentile as a distribution threshold to select the best brokers, bridges, and bottlenecks (above red line). The Y-axis in the figure is the node attribute metric. The bridgeness centrality (“nodes bridgeness”) of a node is the product of the betweenness centrality and the bridging coefficient, which measures the extent to which a node or an edge is located between well-connected regions. The “nodes brokering” is the measure that represents nodes with high betweenness centrality and lower clustering coefficient. The “nodes bottlenecks” is the measure that represents nodes with high betweenness centrality.
