# Supplementary figures and images for: Whole-exome sequencing in obsessive-compulsive disorder identifies rare mutations in immunological and neurodevelopmental pathways
Source: Transl Psychiatry. 2016 Mar 29;6(3):e764–. doi: 10.1038/tp.2016.30 (PMC4872454; doi:10.1038/tp.2016.30)

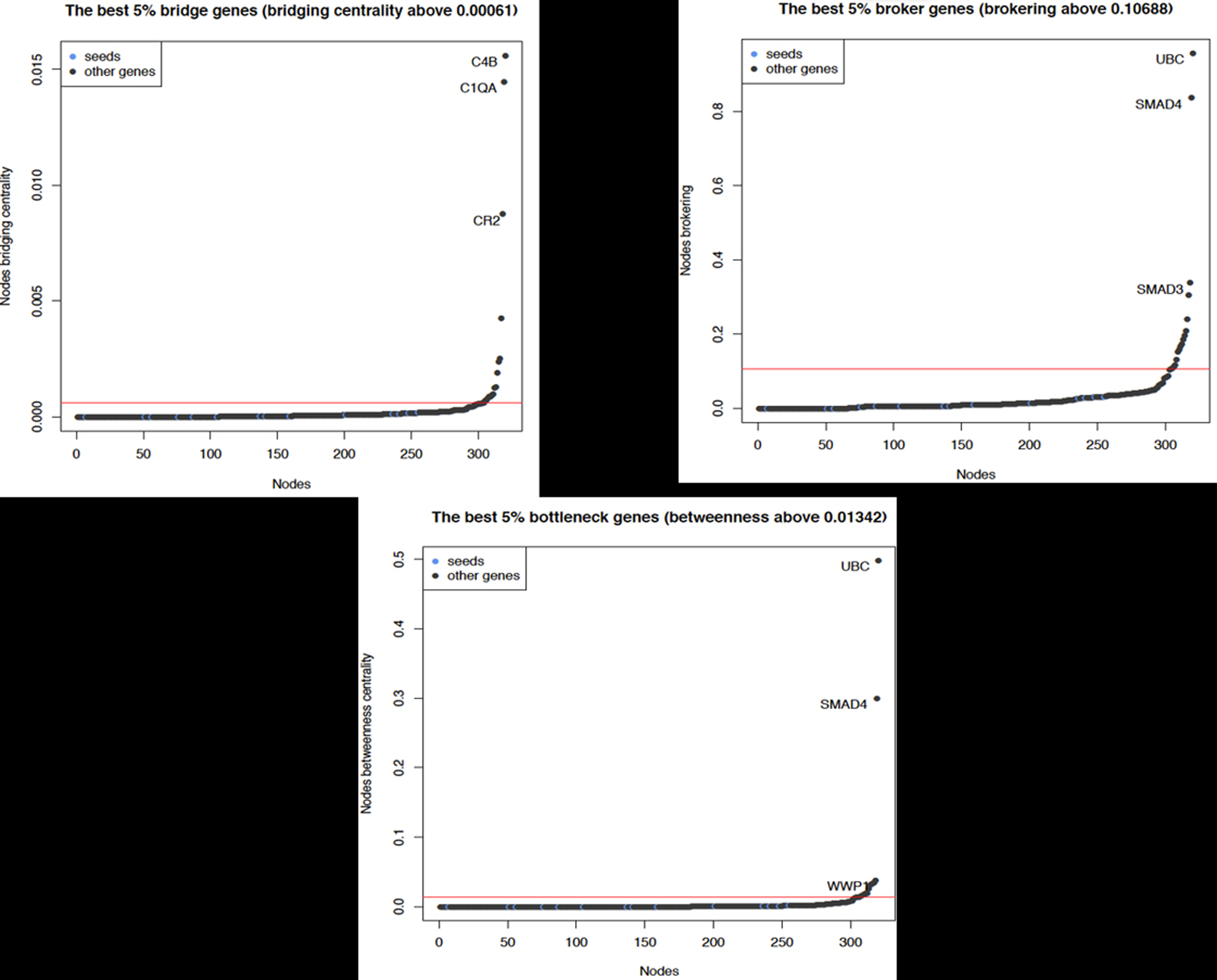

Supplement: Supplementary Figure [file tp201630x4.tif]
